# Bioactivity and phenolics profile of aqueous and ethyl acetate extracts of *Satureja kitaibelii* Wierzb. ex Heuff. obtained by ultrasound-assisted extraction

**DOI:** 10.1038/s41598-022-25668-3

**Published:** 2022-12-08

**Authors:** Kristina Gopčević, Slavica Grujić, Jelena Arsenijević, Ana Džamić, Ivona Veličković, Lidija Izrael-Živković, Ana Medić, Jelena Mudrić, Marina Soković, Ana Đurić

**Affiliations:** 1 Institute of Chemistry in Medicine “Prof. Dr. Petar Matavuljˮ, Faculty of Medicine, University of Belgrade, Višegradska 26, Belgrade, 11000 Serbia; 2 Institute of Botany and Botanical Garden Jevremovac, Faculty of Biology, University of Belgrade, Studentski Trg 16, Takovska 43, Belgrade, 11000 Serbia; 3 Department for Pharmacognosy, Faculty of Pharmacy, University of Belgrade, Vojvode Stepe 450, Belgrade, 11000 Serbia; 4 Institute for Medicinal Plant Research “Dr. Josif Pančić”, Tadeuša Košćuška 1, Belgrade, 11000 Serbia; 5 Institute for Biological Research “Siniša Stanković” – National Institute of Republic of Serbia, University of Belgrade, Bulevar Despota Stefana 142, Belgrade, 11000 Serbia; 6 Institute of Oncology and Radiology of Serbia, Pasterova 11, Belgrade, Serbia

**Keywords:** Plant breeding, Biochemistry

## Abstract

The aim of the study was to investigate the biological activity and chemical composition of *Satureja kitaibelii* Wierzb. ex Heuff. LC–PDA/MS analyses for the aqueous extracts (A1-stem, leaves and flowers, A2-leaves and flowers) and ethyl-acetate extracts (E1-stem, leaves and flowers, E2-leaves and flowers) obtained by ultrasound-assisted extraction enabled the identification of thirty-four compounds. Quantitative analysis revealed that the aqueous extract obtained from leaves and flowers was the richest in total phenolic acids (65.36 mg/g) and flavonoids (21.17 mg/g). The total polyphenol content was the highest in the aqueous extract obtained from leaves and flowers (274 ± 2.4 mg Gallic Acid equivalents/g). The best antioxidant activity was observed for the same extract using the DPPH (SC50 20 ± 10 µg/mL), ABTS (2.834 ± 0.02 mg Ascorbic Acid/g), FRAP (1.922 ± 0.03 mmol Fe^2+^/mg), and total reducing power tests (16.4 ± 1.0 mg Ascorbic Acid/g). Both ethyl acetate extracts were the most active against strains of *Bacillus cereus* and *Micrococcus flavus* (MIC 1.70–1.99 mg/mL and 1.99–3.41 mg/mL, respectively). They were more efficient against *Aspergillus ochraceus* (MFC 0.86 mg/mL) and towards HeLa cell lines. All the obtained results implied the good potential of the investigated extracts to be used as effective preservatives and functional ingredients in food products and dietary supplements.

## Introduction

*Satureja kitaibelii* Wiersb. ex Heuff. is an annual, aromatic species that grows in eastern Serbia on warm limestone rocks. It is also referred to as *S. montana* subsp*. kitaibelii* (Wierzb ex Heuff.) P.W. Ball or *S. montana* var*. kitaibelii* (Wierzb. ex Heuff.) Nyman^1^. This is one of the most popular species in Serbian traditional herbal medicine, usually consumed as winter savory ("*rtanj tea*")^2^ for treatment of respiratory diseases, urinary problems and digestive disorders, externally for skin and mucous inflammation, and as an aphrodisiac^3–5^. Essential oil analysis of *S. kitaibelii* and its antimicrobial activity was well documented^6–12^, while data on the composition and bioactivity of *S. kitaibelii* extracts are scarce. It was confirmed that aqueous-alcohol and ethanol extracts were rich in phenolic compounds and exhibited good antioxidant activity^13–15^. Methanol extract exhibited modest antimicrobial activity and antitumor activity against malignant cells^3,16^. The antimicrobial activity of aqueous extract obtained using various extraction techniques was also proven^16,17^. The examination of time-kill kinetics of *S. kitaibelii* subcritical aqueous extract showed its effects on the viability of the tested sensitive microorganisms in the initial contact phase, while prolonged contact time was needed for bacteriostatic effects^17^. However, there are still insufficient data on the chemical composition and biological activities of aqueous and ethyl acetate extracts of *S. kitaibelii* obtained using ultrasound-assisted extraction, as well as on the distribution of biactive molecules in their different aerial parts. A different distribution of bioactive components and antioxidants in different parts of plants is well documented^18–21^. In the current study, we analyzed different aerial parts of the plant in order to gain insight into the content and distribution of bioactive ingredients. The aim of the present study was to evaluate these extracts as a potential source of valuable phytochemicals and beneficial activity, which might promote *S. kitaibelii* as a functional food ingredient or food additive. In that regard, the examination carried out within the study encompassed the chemical composition, antioxidative, antimicrobial and cytotoxic activities of the ethyl acetate and aqueous extracts of the different aerial plant parts of *S. kitaibelii* obtained using ultrasound-assisted procedure.

## Results

### Phytochemical analysis

LC–PDA/MS analysis of the examined extracts of *S. kitaibelii* revealed the presence of phenolic acids and flavonoids, as well as of jasmonic acid derivatives and a diterpene. The obtained chromatographic and spectral data, *i.e.* retention times (*R*t) on UV (350 nm) and MS chromatograms, *λmax* (nm), ESI–MS data (*m/z*), peak assignments, as well as the occurrence and content of the compounds in the extracts, are presented in Table 1. Chromatograms of the extracts are shown on Supplementary Fig. 1.

**Table 1:** Results of qualitative and quantitative LC–PDA/MS analysis of aqueous and ethyl acetate extracts of stems, leaves and flowers (A1 and E1, respectively) and leaves and flowers (A2 and E2, respectively) of *S. kitaibelii.*

Peak#	*R*_*t*_ (UV)	*R*_*t*_ (MS)	Assignement	Spectral data		Content (mg/g of dry extract)				Reference
				*λ*_*max*_ (nm)	ESI–MS data (m/z)	A1	A2	E1	E2	
***Jasmonic acid derivatives***										
2	9.92	10.01	12-Hydroxyjasmonic acid 12-O-hexoside	228	387.2 [M–H]^−^	d.n.d	d.n.d	d.n.d	d.n.d	Pacifico et al., 2015^L^; Moreira et al., 2020^S^
20	15.48	15.59	12-O-(Caffeoylhexosyl)-jasmonate	230, 294, 326	549.2 [M–H]^−^	n.d	d n.d	d.n.d	d.n.d	Pacifico et al., 2015^L^;
***Phenolic acids***										
			**Simple HCA**							
3	11.02	11.17	Caffeic acid * ^b^	296, 326	179.0 [M–H]ˉ	tr	2.354 ± 0.050	0.184 ± 0.007	0.453 ± 0.019	Moreira et al., 2020^S^; Tsimogiannis et al., 2017^S^
12	13.72	13.75	*p*-Coumaric acid * ^b^	312	163.0 [M–H]ˉ	**1.223 ± 0.078**	n.d	n.d	n.d	Boroja et al., 2018^S^; Čakar et al., 2018^S^
			**Caffeoylquinic acids**							
1	9.49	9.61	Chlorogenic acid * ^a^	298, 326	353.1 [M–H]ˉ, 191.0	0.127 ± 0.025	n.d	**1.317 ± 0.005**	n.d	Boroja et al., 2018^S^; López-Cobo et al., 2015^S^
4	11.48	11.60	Cynarin * ^a^	298, 324	515.1 [M–H]ˉ, 353.1	n.d	n.d	0.535 ± 0.001	n.d	López-Cobo et al., H]^−^ 2015^S^
15	14.38	14.50	Dicaffeoylquinic acid isomer ^a^	n/a	515.1 [M–H]^−^, 353.1	n.d	n.d	0.682 ± 0.022	n.d	López-Cobo et al., 2015^S^
18	15.16	15.27	3,5-Dicaffeoylquinic acid * ^a^	296, 328	515.0 [M–, 353.0	0.333 ± 0.360	n.d	**1.822 ± 0.016**	n.d	López-Cobo et al., 2015^S^
			**HCA dimers**							
21	15.90	16.01	Rosmarinic acid * ^b^	294, 328	359.0 [M–, 197.1, 161.0	tr.	**15.046 ± 2.545**	**1.755 ± 0.039**	**5.719 ± 1.045**	Damašius et al., 2014^S^; Moghadam et al., 2015^S^; Boroja et al., 2018^S^; Gomes et al., 2020^S^
			**HCA trimers**							
7	12.96	13.08	Salvianolic acid K/isomer ^b^	284	555.1 [M–H]ˉ, 357.0	tr	0.210 ± 0.163	tr	tr	Celano et al., 2017^L^
19	15.24	15.35	Salvianolic acid A/isomer ^b^	288, 330	493.1 [M–H]ˉ, 331.1	tr	2.305 ± 0.439	n.d	n.d	Tsimogiannis et al., 2017^S^; Moreira et al., 2020^S^; Gomes et al., 2020^S^
			**HCA tetramers**							
22	16.69	16.74	Clinopodic acid I ^b^	286, 330	717.2 [M–H]ˉ, 519.1, 357.0	n.d	2.077 ± 0.809	n.d	tr	Moghadam et al., 2015^S^
24	17.01	17.12	Salvianoilc acid E/L isomer ^b^	286, 330	717.1 [M–H]ˉ, 519.1, 339.0	n.d	2.886 ± 0.175	n.d	tr	Chen et al., 2011^L^; Celano et al., 2017^L^
			**HCA hexamers**							
25	17.79	17.90	Clinopodic acid O ^b^	286, 330	1075.2 [M–H]ˉ, 519.0, 339.0	tr	**35.627 ± 2.533**	0.233 ± 0.007	**1.164 ± 0.205**	Moghadam et al., 2015^S^
27	18.44	18.55	Clinopodic acid K ^b^	286, 330	1075.2 [M–H]^−^, 357.0	n.d	3.137 ± 0.116	n.d	tr	Moghadam et al., 2015^S^
30	19.30	19.33	Hydroxycinnamic acid hexamer ^b^	286, 330	1075.1 [M–, 555.1, 519.1, 357.1	n.d	1.721 ± 0.384	n.d	tr	Celano et al., 2017^L^; Pacifico et al., 2015^L^
*Total phenolic acids*			*1.68*	*65.36*	*6.53*	*7.34*				
***Flavonoids***										
			**Flavonoid glycosides**							
5	11.56	11.63	Luteolin 7-*O*-diglucuronide * ^c^	254, 266, 348	637.1 [M–, 351.0, 285.0	**2.569 ± 0.089**	**7.955 ± 0.699**	tr	tr	Gomes et al., 2020^S^; Marczak et al., 2010
6	12.77	12.91	Apigenin dihexuronide ^b^	268, 334	621.1[M–H]ˉ, 351.0, 269.0	0.091 ± 0.005	tr	n.d	n.d	Marczak et al., 2010
8	13.18	13.25	Luteolin 7-*O*-rutinoside * ^b^	254, 268, 348	593.2 [M–H]ˉ, 285.0	tr	tr	0.048 ± 0.036	tr	Tsimogiannis et al., 2017^S^;
9	13.21	13.34	Isoquercitrin * ^b^	256, 266, 354	463.1 [M–H]ˉ	n.d	n.d	n.d	0.252 ± 0.051	Boroja et al., 2018^S^;
10	13.23	13.33	Luteolin caffeoyl-dihexuronide ^b^	252, 270sh, 338	799.1 [M–H]ˉ, 513.1, 285.0	0.674 ± 0.800	5.732 ± 0.401	n.d	n.d	Moghadam et al., 2015; Marczak et al., 2010; Stochmal and Oleszek, 2007
11	13.43	13.55	Luteolin sinapoyl-dihexuronide ^b^	270, 336	843.2 [M–H]ˉ, 557.2, 285.0	n.d	2.312 ± 0.331	n.d	n.d	Marczak et al., 2010; Stochmal and Oleszek, 2007
13	13.95	14.06	Luteolin 7-*O*-glucuronide * ^b^	254, 266, 348	461.0 [M–H]ˉ, 285.0	**0.778 ± 0.087**	3.886 ± 0.607	0.053 ± 0.005	0.136 ± 0.051	Moghadam et al., 2015^S^; Celano et al., 2017^L^
14	14.25	14.35	Apigenin deoxyhexosyl-hexoside ^b^	268, 332	577.2 [M–H]ˉ, 269.0	n.d	tr	tr	tr	Hossain et al., 2010^L^
16	14.43	14.56	Luteolin *p*-coumaroyl-dihexuronide ^b^	270, 326	783.1 [M–H]^−^, 285.0	0.229 ± 0.049	1.288 ± 0.100	n.d	n.d	Marczak et al., 2010; Stochmal and Oleszek, 2007
17	14.75	14.86	Diosmin * ^b^	n/a	607.1 [M–, 299.0, 284.0	tr	n.d	tr	tr	Celano et al., 2017^L^
23	16.77	16.90	Me-apigenin deoxyhexosyl-hexoside ^b^	268, 332	591 [M–, 283, 268	0.038 ± 0.007	2.384 ± 0.664	1.178 ± 0.123	**1.258 ± 0.370**	López-Cobo et al., 2015^S^; Šimunović et al., 2020^S^; Marin et al., 2001^S^
26	18.25	18.36	Me-apigenin hexoside ^b^	332	445.0 [M–H]ˉ, 283.0, 268.0	tr	n.d	n.d	n.d	Park et al., 2019^L^
			**Flavonoid aglycons**							
28	18.71	18.94	Eriodyctiol * ^d^	288	287.1 [M–H]ˉ	n.d	n.d	tr	tr	Tsimogiannis et al., 2017^S^;
29	19.26	19.38	Luteolin * ^d^	348	285.0 [M–H]ˉ	tr	n.d	0.105 ± 0.005	tr	Tsimogiannis et al., 2017^S^; Boroja et al., 2018^S^
31	21.33	21.39	Naringenin * ^d^	288	271.0 [M–H]ˉ	n.d	n.d	tr	tr	Tsimogiannis et al., 2017^S^; Boroja et al., 2018^S^; Čakar et al., 2018^S^
32	21.61	21.72	Apigenin * ^d^	336	269.0 [M–H]ˉ	n.d	n.d	0.127 ± 0.007	tr	López-Cobo et al., 2015^S^; Boroja et al., 2018^S^
34	26.02	26.13	Genkwanin * ^d^	334	283.0 [M–H]ˉ, 268.0	tr	n.d	0.291 ± 0.008	tr	Hossain et al., 2010^L^
*Total flavonoids*				*4.06*	*21.17*	*6.53*	*1.65*			
***Diterpenes***										
33	24.35	24.47	Rosmanol / isomer	280, 328	345.1 [M–H]^−^, 283.0	n.d	n.d	d.n.d	d.n.d	Celano et al., 2017^L^; Hossain et al., 2010^L^

HCA, hydroxycinnamic acid; n/a, not available; n.d., not detected; d.n.d., detected, but not determined; tr., trace.

*Constituent identified by comparison with the standard compound; ^S^previously detected in a plant belonging to the genus *Satureja*; ^L^previously detected in a plant belonging to the family Lamiaceae.

^a^Expressed as chlorogenic acid equivalents; ^b^expressed as rosmarinic acid equivalents; ^c^expressed as luteolin 7-*O*-glucoside equivalents; ^d^expressed as luteolin equivalents. Major constituents in extracts are marked in bold figures.

### Qualitative composition of the extracts

Fourteen out of 34 identified compounds belonged to phenolic acids, all being derivatives of hydroxycinnamic acid (HCA), including simple acids, depsides of quinic acid, and especially HCA oligomers. Among the latter, the most abundant were dimeric rosmarinic acid (**21**) and hexameric clinopodic acid O (**25**) (Supplementary Fig. 1). Moreover, two trimers (**7** and **19**), two tetramers (**22** and **24**), and two additional hexamers of HCA (**27** and **30**) were detected in the extracts. The majority (14) of eighteen identified flavonoids were flavones, *i.e.* aglycons luteolin (**29**), apigenin (**32**), and genkwanin (**34**), as well as glycosides of luteolin (**5**, **8**, **10**, **11**, **13**, and **16**) and apigenin (**6** and **14**) and their methylated derivatives (**17**, **23**, and **26**) (Table 1). The MS data indicated that the sugar moieties of glycosides were either monosaccharide (hexose or hexuronic acid) or disaccharide units (composed of two hexuronic acids or deoxyhexose and hexose). In addition, among luteolin glycosides, three were identified as those acylated with HCAs (**10**, **11**, **16**) based on their UV and MS spectral characteristics^31–33^. In the current study, we also detected the presence of one flavonol glycoside (**9**), two flavanone aglycons (**28**, **31**), two hexosyl jasmonic acid derivatives (**2**, **20**) and one diterpene (**33**) in the *S. kitaibelii* extracts.

**Figure 1:**
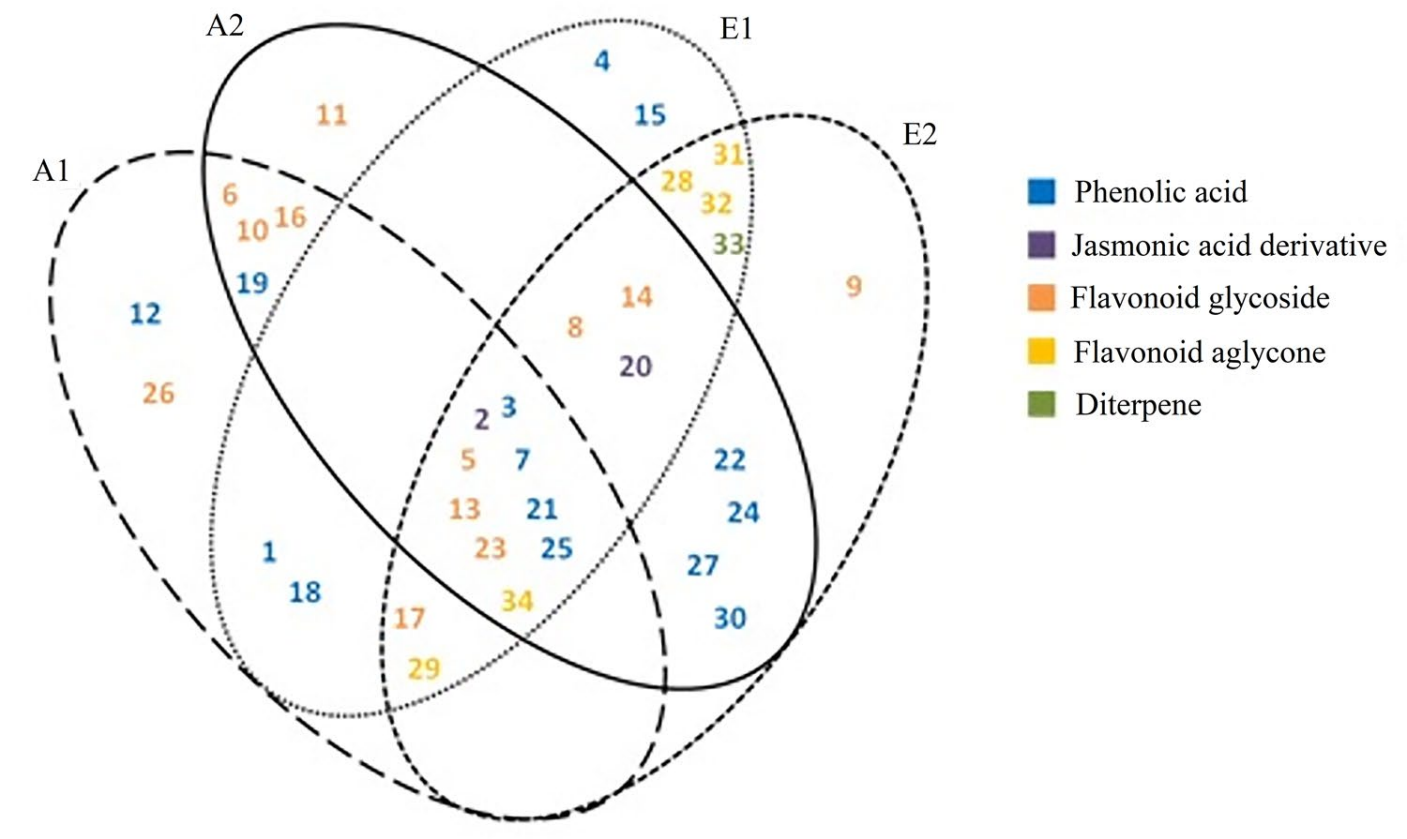
Distribution of the identified constituents (**1**–**34**) in aqueous and ethyl acetate extracts of stems, leaves and (**29**), Hydroxycinnamic acid hexamer (**30**), Naringenin (**31**), Apigenin (**32**), Rosmanol /isomer (**33**), Genkwanin (**34**).

Figure 1 shows the distribution of the identified constituents between the extracts. Nine compounds were common to all extracts: 12-hydroxyjasmonic acid 12-*O*-hexoside (**2**), caffeic acid (**3**), luteolin 7-*O*-diglucuronide (**5**), salvianolic acid K/isomer (**7**), luteolin 7-*O*-glucuronide (**13**), rosmarinic acid (**21**), Me-apigenin deoxyhexosyl-hexoside (**23**), clinopodic acid O (**25**), and genkwanin (**34**). By sharing 18 common compounds, ethyl acetate extracts E1 and E2 were the most similar in terms of qualitative composition compared to other extracts, emphasizing the selectivity of the extracting solvent, while the aqueous extracts shared 13 compounds.

### Quantitative composition of the extracts

Quantitative chromatographic analysis revealed that extract A2 was the richest among the assayed extracts regarding the content of two major groups of metabolites, *i.e.* phenolic acids and flavonoids (Table 1, Supplementary Fig. 1), while other extracts contained substantially lower amounts of these specialized metabolites. The lowest amount of phenolic acids was observed in the extract A1, with HCA oligomers present only in trace amounts, while E2 was the poorest regarding flavonoids.

In the extract A2, the most abundant compounds were clinopodic acid O, rosmarinic acid, and luteolin 7-*O*-diglucuronide (35.627 mg/g, 15.046 mg/g and 7.955 mg/g, respectively). Rosmarinic acid and clinopodic acid O, together with Me-apigenin deoxyhexosyl-hexoside, were the dominant constituents of the extract E2 (5.719 mg/g, 1.164 mg/g, and 1.258 mg/g, respectively). In the extract A1, luteolin 7-*O*-diglucuronide, *p*-coumaric acid, and luteolin 7-*O*-glucuronide dominated over other compounds (2.569 mg/g, 1.223 mg/g, and 0.778 mg/g, respectively), while E1 contained 3,5-dicaffeoylquinic acid, rosmarinic acid, and chlorogenic acid as the major constituents (1.822 mg/g, 1.755 mg/g, and 1.317 mg/g, respectively). Moreover, the abundance of every single caffeoylquinic acid was the highest in the extract E1.

#### Total phenolics content

Differences between extracts in their total phenol content that were noticed due to different polarity of extracting solvents and using different plant parts and results are summarized in Table 2.

**Table 2:** Total phenols contents of aqueous and ethyl acetate extracts of stems, leaves and flowers (A1 and E1, respectively) and leaves and flowers (A2 and E2, respectively) of *S. kitaibelii* and comparison with relevant studies.

Total phenols (mg GA/g)						
References	Species	Extracts				Extraction medium
		A1	A2	E1	E2	
This study	*S. kitaibelii* Wiersb. ex Heuff	164 ± 1.80	274 ± 2.4	44.5 ± 2	51.5 ± 1.2	Water EtOAc
Trifonova et al., 2021	*S. kitaibelii* Wierzb. ex Heuff. (flowering stems)	160.10 mg GA/g				Water
Lopez-Cobo et al., 2015	*S. montana* subsp. *kitaibelii* (Wierzb. ex Heuff.) P.W. Ball	25.82 mg GA/g				Aqueous MeOH
Gopčević et al., 2019	*S. kitaibelii* Wiersb. ex Heuff	105.06 ± 11.32–158.54 ± 14.15 mg GA/g (stems, leaves and flowers) 158.85 ± 15.02–195.95 ± 21.35 mg GA/g (leaves and flowers)				EtOH
Ćetković et al., 2007	*S. montana* L. subsp. *kitaibelii* (aerial parts)	969.43 µg GA/g 1358.14 µg GA/g,				EtOAc *n*-ButOH
Moreira et al., 2020	*S.montana* L. (leaves)	12–24 mg GA/g				Water
Serrano et al., 2011	*S. montana* L	164.2 mg GA/g, 187.5 mg GA/g				Cold water Hot water
Zeković et al., 2017	*S. montana* L	29.5–74.4 mg GA/g				Water-EtOH
Vladić et al., 2014	*S. montana* L	39–72 mg GA/g				Water- EtOH (20–80%)
Tsimogiannis et al., 2017	*S. thymbra* L. (leaves)	249.5 ± 0.9 mg GA/g 154.0 ± 11.9 mg GA/g 289.3 ± 6.2 mg GA/g				Water EtOAc EtOH
Choulitoudi et al., 2016	*S. thymbra* (leaves)	154.0 mg GA/g				EtOAc

*EtOH* ethanol, *MeOH* methanol, *EtOAc* ethyl acetate; Polarity index: Water − 1; Methanol − 0.762; Ethanol − 0.654, Ethyl Acetate − 0.440.

The TPC has been determined in aqueous and ethyl acetate extracts of *S. kitaibelii* in this work for the first time. The highest content of total phenols was observed in the A2 extract (274 mg GA/g). The TPC was higher in both aqueous extracts (164 and 274 mg GA/g), compared to the TPC of both ethyl acetate extracts (44.5 and 51.5 mg GA/g).

### The antioxidant activity

The antioxidant activity of an extract was determined as free radical scavenging capacity and reducing power and measured by DPPH, ABTS, FRAP, and TRP assays. The results are presented in Table 3.

**Table 3:** Free radical scavenging activity and reducing power of aqueous and ethyl acetate extracts of stem, leaves and flowers (A1 and E1, respectively) and leaves and flowers (A2 and E2, respectively) of *S. kitaibelii.* BHA and L-ascorbic acid were used as standard antioxidants.

Extract	DPPH SC_50_ (µg/mL)	ABTS (mg AA/g)	FRAP (μmol Fe^+2^ /mg)	TRP (mg AA/g)
A1	35 ± 10	2.105 ± 0.05	1.547 ± 0.02	10.5 ± 0.4
A2	20 ± 10	2.834 ± 0.02	1.922 ± 0.03	16.4 ± 1.0
E1	320 ± 30	0.449 ± 0.01	0.257 ± 0.01	7.7 ± 0.4
E2	280 ± 20	0.467 ± 0.19	0.430 ± 0.02	7.19 ± 0.5
BHA	5.43 ± 0.02	–	1.83 ± 0.41	10.97 ± 0.29
*L*-ascorbic acid	3.74 ± 0.12	–	6.30 ± 0.22	–

### Antimicrobial activity of extracts of S. kitaibelii

**Figure 2:**
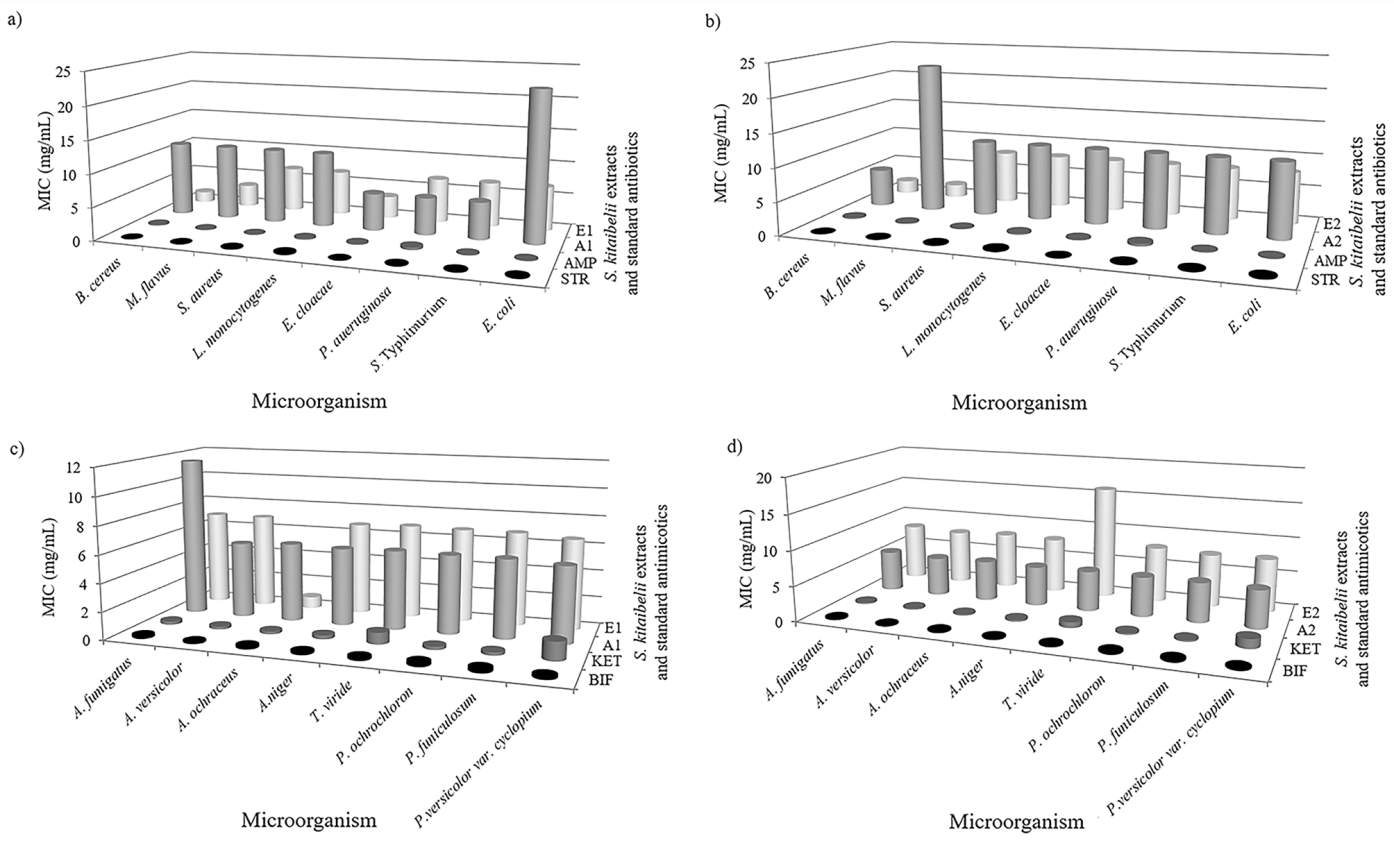
Antibacterial (**a**, **b**) and antifungal activity (**c**, **d**) of aqueous and ethyl acetate extracts of stems, leaves and flowers (A1 and E1, respectively) and leaves and flowers (A2 and E2, respectively) of *S. kitaibelli*. MIC—minimal inhibitory concentration, (mg/mL); STR—streptomycin; AMP—ampicillin; BIF—bifonazole; KET—ketoconazole. Detailed data on all microorganisms are given in the Supplementary Table 2.

Results of the antimicrobial activity of the investigated extracts, presented as a minimal inhibitory concentration for both bacteria and fungi are shown in Fig. 2a–d.

MIC values for bacteria varied from 1.70 to 22.73 mg/mL, while MBC ranged from 3.41 to 45.45 mg/mL (data not shown). Ethyl-acetate and aqueous extract obtained from stem, leaves, and flowers (A1 and E1) were more effective against the majority of the tested bacteria than extracts made of leaves and flowers (A2 and E2). The ethyl acetate extracts (E1 and E2) exhibited lower MICs compared to the aqueous ones, indicating that the antibacterial properties of extracts also depended on solvent polarity which was also confirmed by^13^. Particularly sensitive to ethyl acetate extracts E1 and E2 were *B. cereus* (MIC 1.70 and 1.99 mg/mL, respectively) and *M. flavus* (MIC 3.41 and 1.99 mg/mL, respectively) among gram (+) bacteria. The most affected among tested gram (−) bacteria were *E. cloacae* by ethyl acetate extract of aerial parts (E1) and *E. coli* by ethyl acetate extract of leaves and flowers (E2). However, *E. coli* was the most resistant to the remaining examined extracts (A1, A2, and E1).

#### Cytotoxic activity of S. kitaibelii extracts

The in vitro cytotoxic activity of the investigated extracts is presented as IC_50_ values in Table 4.

**Table 4:** Cytotoxic activity (IC_50_ values) of aqueous and ethyl acetate extracts of stems, leaves and flowers (A1 and E1, respectively) and leaves and flowers (A2 and E2, respectively) of *S. kitaibelii* against human cancer cell lines.

	HeLa	MCF-7	PC-3	MRC-5
**IC**_**50**_** (μg/ml)**				
A1	> 200	188 ± 5.97	> 200	> 200
A2	> 200	182.3 ± 6.06	> 200	> 200
E1	22.26 ± 0.23	168 ± 5.39	74.41 ± 7.9	72.5 ± 5.06
E2	20.42 ± 1.93	155.2 ± 12.36	58.31 ± 1.1	60.92 ± 4.39

IC_50_ values (μg/mL) are expressed as the mean ± SD determined from the results of MTT assay in three independent experiments.

The obtained data suggest that extracts of *S. kitaibelii* showed tumor-selective cytotoxic activity depending on the cell line type and extracting agent. Both ethyl acetate extracts showed stronger cytotoxic effects towards HeLa, PC-3, and MCF-7 cancer cell lines, as well as towards non-malignant cell lines, compared to the aqueous extracts. The HeLa cell line was the most sensitive to the presence of ethyl acetate extracts, PC-3 and healthy MRC-5 cells were moderately sensitive, whereas quite a poor effect was observed towards the MCF-7 cell line. Aqueous extracts A1 and A2 exhibited low cytotoxic activity only towards the MCF-7 cell line (Table 4).

## Discussion

In the current study, phytochemical analysis by LC–PDA/MS was performed to investigate the bioactive compounds present in an aqueous and ethyl acetate extract of *Satureja kitaibelii* obtained from different aerial parts by ultrasound assisted extraction. LC/PDA-MS analysis revealed the presence of numerous compounds, some of which were identified for the first time. Derivatives of HCA are common for plants belonging to the family Lamiaceae. Rosmarinic acid is often one of the major polyphenol constituents in aerial parts of polar extracts of these plants, including plants of the genus *Satureja*^34–38^. Besides rosmarinic acid, Lamiaceae species produce higher HCA oligomers which contain up to 8 monomer units^33,39^. In several studies, trimeric^33,40^ and tetrameric oligomers^41–43^ were detected in the leaf or herb extracts of *Satureja* species, including previously analyzed ethanol extracts of *S. kitaibelii*^15^. On the other hand, to the best of our knowledge, clinopodic acids O and K, *i.e.* HCA hexamers, were reported only in the extracts of *S. biflora*, *S. hortensis* L., and previously analyzed ethanol extracts of *S. kitaibelii*^15,33,44^, in addition to a few other Lamiaceae species^45,46^. In the previously analyzed polar extracts of savory species, in addition to various flavone glycosides, a limited number of flavonol and flavanone glycosides were found, which is consistent with the currently analyzed aqueous extracts of *S. kitaibelii*. The presence of mono- and diglucuronides, glucosides, diglucosides, and rutinosides of luteolin, apigenin, and their hydroxylated and methylated derivatives was confirmed in several studies^40,43,47,48^. On the other hand, as far as we are aware, the presence of acylated glycosides, such as those detected in the present work, was rarely reported in plants of the genus *Satureja*^33^. The extraction conditions, as well as species of specific composition, might influence such behavior.

Several more observations could be made on the distribution of compounds concerning the starting plant material and solvent used for the preparation of the extracts. With the exception of luteolin glucuronides (**5** and **13)**, which were detected in all extracts (although in low amounts in ethyl acetate extracts), other detected flavonoid hexuronides (**6**, **10**, **11**, **16**) were found exclusively in aqueous extracts, which corresponds to their high hydrophilicity. In that regard, rosmanol/isomer (**33**) and all flavonoid aglycons (**28**, **29**, **31**, **32**, and, **34**), *i.e.* compounds of lower hydrophilicity, were common for both ethyl acetate extracts regardless of the starting plant material. It is also worth noting that, except for clinopodic acid O which was present in all extracts, all four other HCA tetramers and hexamers were detected only in extracts A2 and E2. On the other hand, caffeoylquinic acids were detected in A1, E1, or both. The presented results indicate that HCA oligomers are predominantly located in the leaves and flowers of *S. kitaibelii*, whereas caffeoylquinic acids are distributed mainly in the stems. However, an especially high content of rosmarinic acid was previously determined in flowers of *Glechoma hederacea* L. (Lamiaceae) in comparison to leaves and stems. At the same time, the highest amount of chlorogenic acid was found in the leaves of this herb (even though its distribution varied very slightly)^49,50^. All this suggests that the proportion of leaves, flowers, and stems in the starting material played an important role in the process of extraction in the current study. The obtained results indicate that the composition of extracts is highly influenced by both the starting plant material and the extracting agent. This is to some extent in contrast with the previous findings, that the contents of rosmarinic acid and clinopodic acid O in the ethanol extracts of leaves + flowers or stems + leaves + flowers of *S. kitaibelii* were much more dependent on the application of ultrasound during the extraction than on the starting plant material^15^. However, the solubility and extractability of metabolites present in *S. kitaibelii* in ethanol, which was used in the previous study, and water and ethyl acetate are much different, and thus substantially control the process of extraction.

The TPC values measured in this work (from both aqueous and ethyl acetate extracts) were comparable with some among those that were previously obtained^14,15,51^ (Table 2) but higher than the values for EtOAc and n-ButOH extracts of aerial parts in^13^.

The results of both assays for free radical scavenging ability of the investigated extracts of *S. kitaibelii* indicate the influence of the solvents used in this activity. Namely, aqueous extracts showed higher antioxidant activity than ethyl acetate ones, which is well correlated with the determined TPC. The difference in the activity of the aqueous and ethyl acetate extracts is a consequence of different compositions of obtained extracts in terms of the components that are extracted depending on the different properties of the extractants (Table 1). The aqueous extracts, especially from leaves and flowers (A2), were the most powerful in the neutralization of DPPH free radicals (IC_50_ 20 µg/mL), which follows the most favorable composition of this extract in comparison to other investigated extracts concerning radical scavenging features of the present compounds. Ethyl acetate extracts of *S. kitaibelii* expressed lower values as compared to the aqueous ones (IC_50_ 280–320 µg/mL). Those data are in agreement with the reported results of^52^, where *S. kitabelii* hot and cold aqueous extracts showed IC_50_ between 9.75–27.92 µg/mL.

Lopez-Cobo et al.^14^ presented results of DPPH activity of 116.36 µg/mL for 70% methanol extract. Ethanol extracts of *S. kitaibelii* aerial parts showed higher SC_50_ values—102.24 and 106.94 µg/mL^15^. Aqueous extracts obtained with ultrasonic extraction with a gradual increase in temperature show IC_50_ value of 0.087 mg/mL^51^. The scavenging effect of both aqueous extracts of *S. kitaibelii* on the ABTS was also more pronounced compared to the ethyl acetate. Aqueous extracts from leaves and flowers showed the strongest free ABTS radicals scavenging ability (2.834 mg AA/g), with values that are comparable to those obtained by^51^. To our knowledge, there is no comparable data on this activity of *S. kitaibelii* extracts.

Likewise, aqueous extracts showed better results on Fe^3+^ reducing activity, measured by the FRAP assay, but they are significantly lower than the values obtained in^52^: (221.74 and 271.88 µmol Fe/g). The highest reducing capacity was showed by an aqueous extract of leaf and flower (16.4 mg AA/g). TRP assay confirmed the results obtained from FRAP. There are no literature data to compare these values.

HCA oligomers, especially rosmarinic acid, were identified in considerable amounts in the examined extracts. Rosmarinic acid shows good antioxidant activity: the value obtained from FRAP was 16.54 mmol Fe^2+^/g and from DPPH assay SC_50_ 3.90 μg/mL^16^.

In a limited number of studies, the activity of HCA oligomers was examined mainly in vitro, and less frequently in vivo. Good antioxidant activity was found for several higher oligomers^53,54^, as along with the ability to inhibit hyaluronidase^55,56^, the ability to inhibit metalloproteinase^51^, the ability to inhibit lipoxygenase^54^, antimicrobial activity^57^, anticancer activity^58^ etc. Antioxidant activity of another phenolic acid present especially in A2, the caffeic acid (CA), could be related to its iron-chelating property through the formation of iron-CA complexes inhibiting Fenton-induced oxidative damage by preventing the formation of free hydroxyl radicals^59^. Furthermore, good antioxidant activity was confirmed for the majority of other constituents. For example, chlorogenic acid (CGA), the most abundant isomer among caffeoylquinic acid in the E1 extract of *S. kitaibelii*, is an important and biologically active polyphenol, playing several important and therapeutic roles such as free radicals scavenger, antioxidant activity, antimicrobial, and many others^60–63^. Flavonoid luteolin and its glycosides possess a variety of pharmacological activities, including antioxidant, anti-inflammatory, antimicrobial, and anticancer activities^17,64^.

Although the antimicrobial activity of the essential oil of different *Satureja* species is well-known, the reports about the antimicrobial activity of extracts are limited. For the evaluation of antibacterial activity, this study used the bacteria causing foodborne diseases and human infections, which are particularly serious in hospitals. Some of them, such as *P. aeruginosa*, *S. aureus*, and *E. coli* are pan-drug-resistant and thus often associated with nosocomial infections. The need for finding new alternative antibacterial agents is therefore quite urgent^65^. The antibacterial effects of *S. kitaibelii* petroleum ether, chloroform, ethyl acetate, and *n*-butanol extracts were examined and the obtained MIC values ranged from 10 to > 100 mg/mL^13^.

The effects of *S. kitaibelii* methanol extract were examined in^3^ and the found MICs (0.32 to 1.25 mg/mL) and MBCs (2.50 to 5.00 mg/mL) were significantly lower than in the current study. The antibacterial activity of the subcritical water extract of *S. kitaibeli* was estimated in^17^ and the obtained MIC values ranged from 1.04 to > 33.3 mg/mL. Unlike in^17^, the results presented herein indicated no selectivity of *S. kitaibelii* extracts among gram (−) and gram (+) bacteria, which is congruent with the results of other researchers^3^.

The microfungi chosen for the evaluation of antifungal effects of *S. kitaibelii* extracts cause plant infections and deterioration of different materials and stored foods. However, the tested microfungi could be potentially hazardous for human health due to the production of mycotoxins, causing infections that especially affect immunosuppressed patients. The MICs for fungi were from 0.86 to 16.06 mg/mL, while MFCs were between 1.72 and 32.12 mg/mL (data not shown). Similar to antibacterial activity, E1 was more efficient in preventing fungal growth, especially against *A. ochraceus* (MIC/MFC 0.86/1.72 mg/mL). Unlike antibacterial activity, the aqueous extracts were more efficient in inhibiting fungal growth, particularly *A.versicolor*, *A.niger*, and *P. verrucosum* var *cyclopium*. The least potent antifungal agent was E2, especially on *T.viride* (MFC/MIC 32.12/16.06 mg/mL). There is very limited research on the antifungal properties of those *S. kitaibelii* extracts. However, *S. kitaibelii* methanol extract inhibited the growth of 6 micromycetes (MIC varied from 0.16 to 1.25 mg/mL) and two *Candida* species (MIC varied from 0.32 to 0.62 mg/mL)^3^. The anticandidial activity of *S. kitaibelii* aqueous extract was later confirmed in^66^. Moreover, *S.cerevisiae* and *Aspergillus brasiliensis* were also sensitive to *S. kitaibelii* aqueous extract^66^.

The antimicrobial properties of *S.kitaibelii* methanol extract were associated with the presence of rosmarinic acid in^3^, which is contradictory not only to results presented herein since its content was the highest in the low active aqueous extract of aerial parts, but also to the previous report on modest antimicrobial activity of this compound^16^. On the other hand, it was suggested in^13^ that other non-phenolic compounds could contribute to the antimicrobial activity of *S. kitaibelii* extracts. In this study, as positive control weused ampicillin, streptomycin, ketoconazole, and fluconazole, antibiotics/antimycotics that are commonly prescribed for the treatment of diseases caused by tested pathogens. The results presented herein showed lower antibacterial/antifungal activity of *S.kitaibelii* extracts than reference standards, which is in agreement with prior published results^3^. The antimicrobial activities of *S. kitaibelii* extracts were insufficiently studied so far, hence the presented study aimed to screen it using the microdilution method and to enable the basis for further examination using various methods for evaluation of antimicrobial properties to obtain more relevant results. However, the presented results indicate that examined *S.kitaibelii* extracts are rich in phenolics, which suggests that particularly influence on the antimicrobial response of extracts, as well as synergistic effects among them, require further study.

In the current study, both types of extracts contained phenolic compounds which previously showed cytotoxic activity in different assays. Several studies have shown that extracts rich in certain phenolic carboxylic acids and flavonoids exhibit cytotoxic activity against the different cell lines, both malignant and healthy, by various mechanisms^43,59,67–70^. The study^70^ has revealed the potential molecular mechanisms of phenolic acids and flavonoids and suggested great promising effects of phenolic acids and flavonoids against breast, colon, lung, and prostate cancers. On the other hand, extracts investigated in the present study were all abundant with phenolic compounds, but the sensitivity of the tested cell lines (except MCF-7) differed markedly depending on the extracting agent used for extract preparation. Additionally, the extract which was the most abundant with phenolic compounds (A2) was not the most active. The reason for this might lie in some differences in the composition which could not be detected through the applied methods, and in the fact that non-phenolic constituents are responsible for the good cytotoxic activity of ethyl acetate extracts. Furthermore, the polarity of compounds present in ethyl acetate extracts might be beneficial for better availability for the tested cell lines. In congruence with the presented results, less polar extracts, especially ethyl acetate extract of *Origanum majorana* showed more pronounced cytotoxicity towards MDA-MB-231 and HT-29 cell lines (IC50 30.90 ± 1.39 and 50.11 ± 1.44 µg/ml) in comparison to aqueous extract (IC50 69.18 ± 3.10 and 177.82 ± 4.07 µg/ml)^71^.

Following the obtained results on the cytotoxicity of ethyl acetate extracts, in a previous study, methanol extract of *S. kitaibellii* exhibited moderate activity against estrogen-dependent (MDA-MB-361) and estrogen-non-dependent (MDA-MB-453) breast cancer, colon cancer (LS174), and HeLa cell lines, as well as strong cytotoxic activity against human malignant melanoma cells (Fem-x), which was partly correlated with the tested activity of rosmarinic acid^3^. On the other hand, the low cytotoxic potential of aqueous extracts of related species *S.subspicata* and *S.horvatii* was previously noted in human lymphocytes *in vitro*^37^, as well as the low potential of *S. Montana* aqueous and aqueous ethanol extracts with IC_50_ values above 600 μg/ml against Caco-2, TR146 and HeLa cell lines^42^. Moreover, aqueous and DMSO extracts of *S. subspicata* and *S. horvatii* downregulated pro-apoptotic and upregulated anti-apoptotic genes of the Bcl-2, showing anti-apoptotic activity, which authors linked to the previously shown similar effects of rosmarinic acid. Similar anti-apoptotic effects, i.e. increased Bcl-2/Bax ratio, were observed for the methanol extract of *S.hortensis*, which was characterized by a high amount of rosmarinic acis^71^. These data, together with the previously confirmed low genotoxic potential of aqueous extracts of *Satureja* plants and rosmarinic acid as their dominant compound^37^, concur with safe usage of *S. kitaibelii* aqueous extracts, but this certainly needs to be better justified in the future.

## Materials and methods

### Plant material, extraction and LC–PDA/MS analysis

Aerial parts of *Satureja kitaibelii* were collected in August 2015, on Mt. Rtanj N 43.752697 E 21.87479, in eastern Serbia. The herb was collected by the botanist Slavica Grujić, in full compliance with all the rules for collecting herbs (at that time, the Ministry of Environmental Protection did not issue permits for collecting this plant, and the species was placed under protection only a few years ago). The plant was identified by Slavica Grujić, and a voucher specimen was deposited in the Herbarium of the Institute of Botany and Botanical Garden "Jevremovac", Faculty of Biology, University of Belgrade, Serbia (No. 17141, BEOU). To avoid destruction of the species population, only individual specimens were collected. A total of 1 kg of fresh plant material was collected (300 g of dried materials). The plant material was air-dried at room temperature in the shadow, deprived of wooden parts.

The air-dried material (10 g), containing stem, leaves, and flowers (sample 1), or leaves and flowers (sample 2) was extracted with 100 mL distilled water or ethyl acetate (polarity of water and ethyl acetate are 1.000 and 0.228, respectively). The extraction procedure was described previously^15^ and resulted in extracts E1 and E2 (obtained from samples 1 and 2, respectively, ethyl acetate), A1, and A2 (obtained from samples 1 and 2, respectively, aqueous). The obtained dried extracts were refrigerated at 4 °C before use.

Qualitative and quantitative chromatographic analysis of the extracts was carried out exactly as described in Gopcevic et al.^15^, on an Agilent 1260 Liquid Chromatograph equipped with an autosampler, a PDA detector, and coupled with a mass detector Agilent MSD 6100 with an electrospray ionization (ESI) source and a single quadrupole analyzer. Chromatographic separation was performed using a Zorbax SB-aq column (150 × 2.1 mm; particle size 3.5 μm) maintained at 27 °C. Extract solutions (3 μL of 5 mg/mL of ethanol) were injected while the pump was operating at a flow rate of 0.3 mL/min. Gradient elution was performed using mobile phase A—0.1% formic acid, and mobile phase B—acetonitrile, being at 0 min 10% B, 25 min 60% B, 30 min 95% B, and returning to the initial conditions during the following 2 min. Chromatograms were recorded at 210, 280, 320, 350, and 370 nm, whereas ESI MS spectra were obtained in negative mode, at the range 150–1150 m/z. The ion chamber was maintained at 350 °C, with nebulization under a nitrogen flow of 10 L/min, pressure of 30 psi, and capillary voltage of 3500 V. Signals were recorded by applying the fragmentor voltage of 100 V, as well as 250 V to obtain additional fragment ions. The identification of constituents was performed by comparison of ultraviolet (UV), mass spectrometry (MS), and retention time (*R*t) data with those obtained for the standard compounds, as well as tentatively, i.e. by comparison of the obtained UV and MS spectra of constituents with the literature reports.

The quantification of phenolic acids and flavonoids was performed by external calibration after regression and correlation analysis, based on the PDA chromatographic data. Rosmarinic acid and chlorogenic acid were used as standard compounds for the determination of phenolic acids, luteolin 7-*O*-glucoside for flavone glycosides, while flavone aglycons were quantified using luteolin as the standard compound. All standard compounds used in LC–PDA/MS analysis of extracts were purchased from Carl Roth, except luteolin 7-*O*-diglucuronide, which was previously isolated from the aerial parts of *Thymus pannonicus*^22^.

The results of quantitative analysis are expressed as a mean from three independent analysis ± standard deviation. Data on the measuring conditions and obtained calibration curves, as well as on the limits of detection (LOD) and quantitation (LOQ), are presented in Supplementary Table S1.

*Total Phenolic Content.* Total phenolic content (TPC) was determined by the method of Singleton and Rossi^23^ with some modifications. To 0.2 mL of suitably pre-diluted extract, 1.8 mL of dH_2_O and 0.2 mL of Folin-Ciocalteu reagent were added sequentially. After five minutes 2 mL of 7% Na_2_CO_3_ were added, and then the mixture was diluted to up to 5 mL with dH_2_O. The reaction continued in the dark for 90 min. The absorbance was measured at 765 nm against dH_2_O. The standard calibration curve was plotted using gallic acid (1–200 µg/mL). The data were calculated according to gallic acid that was used for preparation of calibration curve.

$$ y = 0.177 + 2.563x;\;{\text{r}}^{{2}} = 0.989; $$

The total phenolic content was expressed as gallic acid equivalents per gram of dry extract (mg GA/g).

### Antioxidant activity

#### Ferric reducing antioxidant power (FRAP) assay

The reducing power of extracts was determined using the ferric reducing ability of FRAP assay by Benzie and Strain^24^. This assay was based on the reducing power of a compound (antioxidant). A potential antioxidant will reduce the ferric ion (Fe^3+^) to the ferrous ion (Fe^2+^); the latter forms a blue complex (Fe^2+^/TPTZ), which increases the absorption at 593 nm. In brief, the FRAP reagent was prepared by mixing acetate buffer (200 µL, pH 3.6), a solution of 20 µL TPTZ and 20 µL FeCl_3_ at 10:1:1 volume ratio. The sample solution (200 µL) and the reagent (2.95 mL H_2_O and 1 mL FRAP) were mixed thoroughly and incubated at 37 °C. Absorbance was taken at 593 nm after 10 min. Standard calibration curve was prepared using different concentrations of FeSO_4_⋅7H_2_O. All solutions were freshly prepared. The results were expressed in µmol Fe^2+^/mg dried extract (µmol Fe^2+^/mg d.e.).

#### Scavenging effect on 2,2-diphenyl-1-picrylhydrazyl radical (DPPH)

Antioxidant activity of *S. kitaibelii* extracts was evaluated by the 2,2-diphenyl-1-picrylhydrazil (DPPH) radical scavenging method by Blois^25^. Extracts were diluted in the appropriate solvent, from which further dilutions were made in methanol and DPPH solution (0.04 mg/mL) was added. Samples were vigorously shaken and left for 30 min at room temperature in the dark. Absorbance was measured at 517 nm. All measurements were carried out in triplicate. Absorbance of the remaining DPPH radical in the sample (A1) was measured on 517 nm. Every sample and positive controls (ascorbic acid and BHA) concentration were done in triplicate and the same was done with blank probes, which were prepared to contain methanol instead of the investigated sample (blank absorbance A_0_).

The percentage of scavenging (RSC %) was calculated, as follows:

$$ {\text{RSC}}\;\left( \% \right) = 100\left( {A0 - A1} \right)/A0 $$

The extract concentration providing 50% of free radical scavenging activity (IC_50_) was calculated from the graph of radical scavenging activity (RSA) percentage against the extract concentration.

### 2-Azino-bis(3-ethylbenzthiazoline-6-sulphonic acid) radical-scavenging activity (ABTS)

The ABTS method, based on the reduction of ABTS^•+^ radical in alcohol solution by antioxidants, was performed according to^26^. The ABTS radical cation was generated by the oxidation of ABTS with potassium persulfate (K_2_S_2_O_8_) and its reduction in the presence of hydrogen donating antioxidants was measured spectrophotometrically at 734 nm. The reaction mixture of 19.2 mg of ABTS was dissolved in K_2_S_2_O_8_ before the experiment. This solution was dissolved in distilled water to adjust working solution absorbance to 0.700 at 734 nm. The sample concentration was 1 mg/mL. The reaction mixture was prepared by mixing 100 µL of test sample and 4 mL ABTS. After 30 min’ incubation at 30 °C in a water bath, absorbance of the mixture was measured at 734 nm. The radical scavenging activity for each extract was determined on the basis of the linear calibration curve of ascorbic acid:

$$ \Delta A = A0 - Ax \;({\text{A}}_{0} - {\text{absorbance}}\;{\text{of}}\;{\text{ABTS}}^{ \bullet + } \;{\text{solution}}\;{\text{and}}\;{\text{solvent}};\;{\text{ A}}_{{\text{x}}} - {\text{the}}\;{\text{absorbance}}\;{\text{of}}\;{\text{the}}\;{\text{sample}}). $$

and expressed as mg ascorbic acid/g of dry extract (mg AA/g d.e.).

*The total reducing power (TRP)***-** It was determined according to the method of Oyaizu^27^. The total reducing power assay measures the electron donating capacity of an antioxidant. The presence of reducers causes the conversion of the Fe^3+^/ferricyanide complex to the ferrous form, which serves as a significant indicator of its antioxidant capacity. The reducing capacity of different extracts was compared with ascorbic acid for the reduction of Fe^3+^ to Fe^2+^. In this assay, the color of the test solution changes to various shades of blue, depending on the reducing power of each compound. Therefore, measuring of the formation of Perls’ Prussian blue at 700 nm can monitor the Fe^2+^ concentration. One mL of the extracts was mixed with 2.5 mL of phosphate buffer (0.2 M, pH 6.6) and potassium ferricyanide K_3_[Fe(CN)_6_] (2.5 mL, 1%). The mixtures were incubated at 50 °C for 20 min, after which trichloroacetic acid (10%, 2.5 mL) was added and the mixture was centrifuged. Finally, the upper layer (2.5 mL) was mixed with distilled water (2.5 mL) and FeCl_3_ (0.5 ml; 0.1%). The absorbance of the solution was measured at 700 nm. The blank was prepared with all the reaction agents without extract. Higher absorbance of the reaction mixture indicated that the reducing power is increased. Ascorbic acid was used as standard. The percent of increase in reduction power was calculated by using the following formula:

$$\mathrm{Increase \,  in \, reduction \, power }=100\left(\frac{Atest}{Ablank}-1\right)$$

where Atest is the optical density of the test solution and Ablank is the optical density of the blank solution. The assays were carried out in triplicate and the results were expressed as mean values ± standard deviations as mg AA/g.

All absorbance readings were performed on UV-2600 UV–VIS spectrophotometer (Shimadzu, Japan). All analyses were done in triplicate.

### Antimicrobial activity

The antibacterial activity was evaluated using a microdilution method as previously described in^28^ towards eight bacteria (in Supplementary Table S2): *Bacillus cereus* (clinical isolate), *Micrococcus flavus* ATCC10240 and *Salmonella* Typhimurium ATCC13311, *Listeria monocytogenes* NCTC7973, *Enterobacter cloacae* ATCC35030, *Pseudomonas aeruginosa* IBRSP001, *Staphylococcus aureus* ATCC6538, and *Escherichia coli* ATCC35210. The following microfungi were used for testing antifungal activity according to^29^ (in Supplementary Table S2): *Aspergillus fumigatus* (human isolate), *A. versicolor* ATCC11730, *A. ochraceus* ATCC12066, *A. niger* ATCC6275, *Trichoderma viride* IAM5061, *Penicillium funiculosum* ATCC36839, *P. ochrochloron* ATCC9112, and *P. verrucosum* var. *cyclopium* (food isolate). The examined extracts of *S. kitaibelii* were dissolved in 15% ethanol. Then, serial dilutions were made using Tryptic soy broth (TSB) (bacteria) and Malt extract broth (MEB) (microfungi) in 96-well microplates with a flat bottom. That was followed by incubation for 24 h at 37 °C with inoculated bacteria and for 72 h at 28 °C with inoculated microfungi. Next, the lowest concentrations that caused visible inhibition of bacterial/microfungi growth observed under binocular microscope were determined and defined as minimal inhibitory concentrations (MICs). MBCs/MFCs were determined by re-inoculation of 10 µL and 2 µL of samples, respectively, into the sterile broth. After incubation for another 24 h at 37 °C (bacteria) and for 72 h at 28 °C (microfungi), the lowest concentrations without visible growth that indicated 99.5% killing of the original inoculums were defined as MBCs/MFCs. Negative control in both assays was 15% ethanol. Streptomycin and ampicillin were used as positive controls for antibacterial activity, while bifonazole and ketoconazole were used for antifungal activity since they are commercially available and commonly used standard reference drugs.

### Cytotoxic activity

*Cell lines.* For the in vitro evaluation of the cytotoxic activity of the tested extracts, the following was used: normal human embryonic lung fibroblast (MRC-5), human cervical adenocarcinoma (HeLa), human breast adenocarcinoma (MCF-7) and human prostate adenocarcinoma from bone metastasis (PC-3) cell lines obtained from the American Type Culture Collection (ATCC, Manassas, VA, USA). All cell lines were grown in RPMI-1640 medium supplemented with 3 mM L-glutamine, 100 μg/mL streptomycin, 100 IU/mL penicillin, 10% heat-inactivated (56 °C) fetal bovine serum and 25 mM Hepes adjusted to pH 7.2 with a bicarbonate solution. The cells were grown at 37 °C in an atmosphere of 5% CO_2_ and humidified air. Cells were obtained from the American Type Culture Collection (Manassas, VA, USA), while RPMI 1640, *L*-glutamine and Hepes were obtained from PAA (Pasching, Austria).

*MTT assay for cell viability.* For the evaluation of cytotoxicity of the tested extracts, the MTT assay (3-(4,5-dimethylthiazol-2-yl)-2,5-diphenyltetrazolium bromide—MTT) was used, described previously in^30^. With this colorimetric assay, reductive activity of the cells is determined through conversion of the yellow tetrazolium dye (MTT) to purple formazan crystals by NAD(P)H-dependent oxidoreductase enzymes, present in viable cells. The degree of formazan concentration in the cells correlates to the degree of light absorption. Cells were seeded into 96-well plates by adding 7,000 cells per well for MCF-7; 5,000 cells per well for MRC-5 and PC-3; and 3,000 cells per well for HeLa. On the next day, after the adhesion, cells were treated with five different concentrations of the tested extracts (final concentration ranged from 12.5 μg/ml to 400 μg/ml). Stock solutions, prepared in dimethyl sulfoxide (DMSO), were diluted with a complete nutrient medium and applied to target cells. The treated cells were incubated for 72 h. After the addition of 20 µL of MTT solution (5 mg/mL in phosphate-buffered saline, PBS), samples were incubated for an additional 4 h at 37 °C in a humidified atmosphere of 5% CO_2_ (v/v). Subsequently, 100 mL of 100 g/L sodium dodecyl sulfate (SDS) were added. The absorbance (A) was measured at 570 nm 24 h later.

$$\mathrm{\%\, cell \, viability}=\frac{\mathrm{mean}\left(\mathbf{A}\mathrm{sample}\right)}{\mathrm{mean}(\mathbf{A}\mathrm{blank})}\mathrm{x}100$$

The obtained value, IC_50_, refers to the concentration of the compound that inhibits cell survival by 50% compared to the control. Each experiment with every cell line was performed in triplicate.

### Statistical analysis

All the results are presented as mean ± standard deviations of three determinations. Statistical analyses were performed using the Student’s *t* test and one-way analysis of variance. Correlation between tested activities of various extracts was established using regression analysis at a 95% significance level (*p* ≤ 0.05). IC_50_ values were calculated by nonlinear regression analysis from the sigmoid dose–response inhibition curves. Statistica 6.0 software (2001, Stat Soft, Tulsa, OK, USA) was used to evaluate the *p* < 0.05 level (regarded as significant) and *p* < 0.01 (highly significant).

All methods were performed in accordance with the relevant guidelines and regulations.

## Conclusion

The results of the current study highlighted the aqueous and ethyl acetate extracts of different aerial parts of *Satureja kitaibelii* Wierzb. ex Heuff. endemic plant as a reach source of active compounds, some of which are now extracted and detected for the first time. The aqueous extract of leaves and flowers was the richest source of two major groups of metabolities, i.e. phenolic acids and flavonoids. Dominant constituents, which could be considered as chemical markers, were hydroxycinnamic acid oligomers, rosmarinic acid and clinopodic acid O. All the examined extracts possessed considerable antioxidant activity, which was confirmed by four different assays. Regarding antibacterial activity, both ethyl acetate extracts showed the best results towards *Bacillus cereus* and *Micrococcus flavus*, whereas in case of other tested bacteria all the investigated extracts exhibited moderate to weak activity. In the presence of ethyl acetate extract of all aerial parts, the most sensitive fungus was *Aspergillus ochraceus*, while all other fungal strains were moderately susceptible to all tested extracts. Ethyl acetate extracts were cytotoxic against all tested cell lines, including healthy ones. On the other hand, aqueous extracts exhibited weak activity only towards MCF-7 cells. All the obtained results suggest a good potential of the investigated, especially aqueous, extracts of *S. kitaibelii* to be used as a source of new pharmaceuticals and healthcare products, as well as effective preservatives and functional ingredients in food products, dietary supplements, and other pharmaceutical products.

## Supplementary Information

Supplementary Information.

## Data Availability

The datasets used and/or analyzed during the current study available from the corresponding author on reasonable request.
