# Supplementary material for: Bioactivity and phenolics profile of aqueous and ethyl acetate extracts of Satureja kitaibelii Wierzb. ex Heuff. obtained by ultrasound-assisted extraction
Source: Sci Rep. 2022 Dec 8;12:21221. doi: 10.1038/s41598-022-25668-3 (PMC9731972; doi:10.1038/s41598-022-25668-3)
Supplement: Supplementary file 1 — Supplementary Information. [file 41598_2022_25668_MOESM1_ESM.pdf]

Bioactivity and phenolics profile of aqueous and ethyl acetate extracts of *Satureja kitaibelii* Wierzb. ex Heuff. obtained by ultrasound-assisted extraction

Kristina Gopčević, Slavica Grujić , Jelena Arsenijević, Ana Džamić, Ivona Veličković, Lidija Izrael-Živković, Ana Medić, Jelena Mudrić, Marina Soković, Ana Đurić

Corresponding author: University of Belgrade, Faculty of Medicine, Institute of Chemistry in Medicine "Prof. dr Petar Matavulj", Višegradska 26, 11000 Belgrade, Serbia kristina.gopcevic@med.bg.ac.rs

Supplementary Table 1. Calibration equations, correlation factors (R^2), limits of detection (LOD) and limits of quantification (LOQ) values for chromatographic analysis of standard compounds. a, Y, peak area; X, concentration of the compound (mg/mL) b, LOD and LOQ are estimated according to the ICH Q2(R1) guideline.

**Standard**	**Measuring wavelength (nm)**	**Regression equation**[a]	R^2	**Linear range (mg/mL)**	**LOD**[b] **(mg/mL)**	**LOQ**[b] **(mg/mL)**
Chlorogenic acid	320	Y=29049·X-0.3	0.9998	0.004-0.073	0.001	0.004
Rosmarinic acid	320	Y=25006·X-55.1	0.9997	0.011-0.540	0.013	0.038
Luteolin 7-*O*-glucoside	350	Y=27990·X-5.2	0.9999	0.001-0.080	0.001	0.004
Luteolin	350	Y=53831·X-31.4	0.9998	0.002-0.172	0.003	0.010

[a], Y, peak area; X, concentration of the compound (mg/mL); [b], LOD and LOQ are estimated according to the ICH Q2(R1) guideline

Supplementary Figure 1. Chromatograms of aqueous and ethyl acetate extracts of stems, leaves and flowers (A1 and E1, respectively) and leaves and flowers (A2 and E2, respectively) of *S. kitaibelii* recorded at 350 nm. The numbering of the constituents corresponds to those in **Table 1**.

Chlorogenic acid (**1**), 12-Hydroxyjasmonic acid 12-O-hexoside (**2**), Caffeic acid (**3**), Cynarin (**4**), Luteolin 7-*O*-diglucuronide (**5**), Apigenin dihexuronide (**6**), Salvianolic acid K/isomer (**7**), Luteolin 7-*O*-rutinoside (**8**), Isoquercitrin (**9**), Luteolin caffeoyl-dihexuronide (**10**), Luteolin sinapoyl-dihexuronide (**11**), *p*-Coumaric acid (**12**), Luteolin 7-*O*-glucuronide (**13**), Apigenin deoxyhexosyl-hexoside (**14**), Dicaffeoylquinic acid isomer (**15**), Luteolin *p*-coumaroyl-dihexuronide (**16**), Diosmin (**17**), 3,5-Dicaffeoylquinic acid (**18**), Salvianolic acid A/isomer (**19**), 12-O-(Caffeoylhexosyl)-jasmonate (**20**), Rosmarinic acid (**21**), Clinopodic acid I (**22**), Me-apigenin deoxyhexosyl-hexoside (**23**), Salvianolic acid E/L isomer (**24**), Clinopodic acid O (**25**), Me-apigenin hexoside (**26**), Clinopodic acid K (**27**), Eriodyctiol (**28**), Luteolin (**29**), Hydroxycinnamic acid hexamer (**30**), Naringenin (**31**), Apigenin (**32**), Rosmanol /isomer (**33**), Genkwanin (**34**).

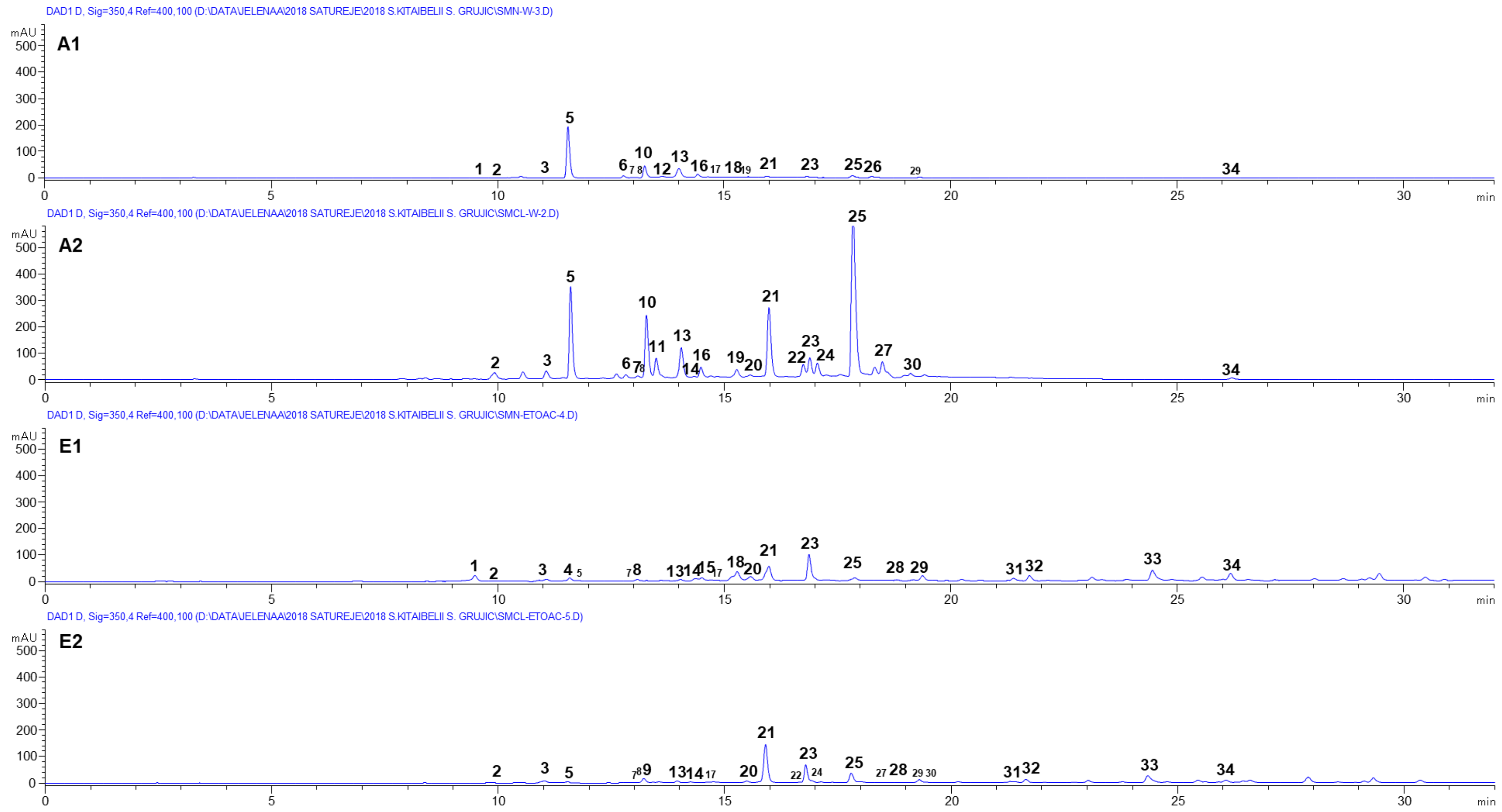

Supplementary Table 2. Bacterial and fungal strains used for the antimicrobial effects examination of aqueous and ethyl acetate extracts of *Satureja kitaibelii*

Bacterial strain	Source	Fungal strain	Source
Bacillus cereus	clinical isolate	*Aspergillus fumigatus*	human isolate
Micrococcus flavus	ATCC10240	*A. versicolor*	ATCC11730
Salmonella Typhimurium	ATCC13311	*A. ochraceus*	ATCC12066
Listeria monocytogenes	NCTC7973	*A. niger*	ATCC6275
Enterobacter cloacae	ATCC35030	*Trichoderma viride*	IAM5061
Pseudomonas aeruginosa	IBRSP001	*Penicillium funiculosum*	ATCC36839
Staphylococcus aureus	ATCC6538	*P. ochrochloron*	ATCC9112
Escherichia coli	ATCC35210	*P. verrucosum* var. *cyclopium*	food isolate

Supplementary Table 3. Raw data on cytotoxic activity of *Satureja kitaibelii* extracts

PC-3
E1/ E2

Measurement count: 1 Filter: 570

	1	2	3	4	5	6	7	8	9
A	0.039	0.04	0.04	0.04	0.039	0.038	0.039	0.039	0.038
B	0.105	0.103	0.103	0.901	0.929	0.945	0.924	0.913	0.947
C	0.114	0.145	0.103	0.903	0.887	0.905	0.9	0.958	0.924
D	0.135	0.121	0.113	0.87	0.904	0.862	0.914	0.895	0.889
E	0.191	0.144	0.145	0.825	0.811	0.804	0.812	0.794	0.812
F	0.215	0.191	0.13	0.49	0.521	0.542	0.672	0.678	0.667
G	0.294	0.274	0.266	0.308	0.314	0.317	0.408	0.384	0.408
H	0.049	0.043	0.044	0.044	0.041	0.043	0.043	0.043	0.041

PC-3
E-Ac1/ E- Ac2

Measurement count: 1 Filter: 570

	1	2	3	4	5	6	7	8	9
A	0.042	0.042	0.041	0.041	0.041	0.04	0.041	0.041	0.04
B	0.11	0.106	0.103	0.956	0.964	0.946	0.961	0.961	0.956
C	0.116	0.107	0.102	0.81	0.766	0.844	0.773	0.796	0.81
D	0.12	0.111	0.109	0.659	0.651	0.71	0.584	0.589	0.641
E	0.129	0.123	0.125	0.314	0.327	0.336	0.255	0.251	0.252
F	0.163	0.148	0.151	0.178	0.206	0.173	0.201	0.19	0.204
G	0.216	0.215	0.214	0.195	0.22	0.203	0.248	0.251	0.249
H	0.04	0.039	0.041	0.039	0.058	0.04	0.04	0.04	0.04

MRC5
E1 / E2

Measurement count: 1 Filter: 570

	1	2	3	4	5	6	7	8	9
A	0.04	0.038	0.04	0.039	0.039	0.04	0.04	0.039	0.04
B	0.091	0.103	0.102	0.864	0.872	0.838	0.865	0.88	0.849
C	0.118	0.11	0.107	0.864	0.798	0.851	0.824	0.842	0.823
D	0.13	0.12	0.122	0.822	0.814	0.812	0.869	0.804	0.82
E	0.156	0.142	0.14	0.795	0.806	0.592	0.954	0.688	0.927
F	0.197	0.19	0.188	0.656	0.754	0.719	0.681	0.733	0.677
G	0.075	0.073	0.073	0.073	0.074	0.073	0.063	0.065	0.063
H	0.041	0.043	0.041	0.041	0.039	0.039	0.039	0.039	0.04

MRC5
E-Ac1/ E-Ac2

Measurement count: 1 Filter: 570

	1	2	3	4	5	6	7	8	9
A	0.043	0.051	0.05	0.043	0.04	0.04	0.04	0.039	0.04
B	0.128	0.108	0.103	0.91	0.902	0.61	0.653	0.878	0.897
C	0.115	0.111	0.101	0.969	0.946	0.923	0.891	0.903	0.914
D	0.122	0.114	0.112	0.553	0.864	0.759	0.624	0.613	0.626
E	0.137	0.127	0.122	0.282	0.276	0.273	0.141	0.166	0.179
F	0.236	0.169	0.137	0.199	0.175	0.179	0.189	0.188	0.189
G	0.232	0.219	0.209	0.232	0.232	0.217	0.235	0.254	0.227
H	0.044	0.042	0.042	0.043	0.045	0.044	0.042	0.044	0.041

HeLa
E1/ E2

Measurement count: 1 Filter: 570

	1	2	3	4	5	6	7	8	9
A	0.04	0.044	0.047	0.044	0.042	0.041	0.042	0.043	0.042
B	0.102	0.109	0.1	0.688	0.685	0.649	0.679	0.709	0.678
C	0.122	0.109	0.11	0.654	0.655	0.652	0.659	0.669	0.669
D	0.133	0.119	0.153	0.608	0.604	0.619	0.655	0.631	0.574
E	0.156	0.126	0.142	0.575	0.585	0.479	0.695	0.572	0.613
F	0.203	0.199	0.176	0.351	0.333	0.296	0.421	0.442	0.449
G	0.292	0.268	0.263	0.274	0.279	0.276	0.336	0.329	0.326
H	0.043	0.041	0.041	0.041	0.042	0.045	0.04	0.04	0.04

HeLa
E-Ac1/ E-Ac2

Measurement count: 1 Filter: 570

	1	2	3	4	5	6	7	8	9
A	0.039	0.04	0.04	0.043	0.042	0.042	0.041	0.04	0.041
B	0.106	0.111	0.103	0.707	0.728	0.744	0.699	0.701	0.713

C	0.099	0.11	0.098	0.475	0.41	0.458	0.329	0.385	0.348
D	0.109	0.143	0.11	0.175	0.175	0.164	0.255	0.179	0.098
E	0.129	0.121	0.148	0.096	0.134	0.149	0.187	0.174	0.155
F	0.192	0.154	0.349	0.178	0.173	0.121	0.251	0.181	0.183
G	0.215	0.205	0.203	0.258	0.3	0.19	0.222	0.287	0.205
H	0.053	0.043	0.042	0.047	0.049	0.048	0.053	0.058	0.046

MCF7
E1/ E2

Measurement count: 1   Filter: 570

	1	2	3	4	5	6	7	8	9
A	0.041	0.04	0.041	0.041	0.041	0.041	0.041	0.04	0.04
B	0.11	0.1	0.096	0.708	0.692	0.708	0.717	0.718	0.716
C	0.123	0.112	0.103	0.7	0.705	0.757	0.646	0.706	0.729
D	0.134	0.12	0.088	0.701	0.655	0.721	0.706	0.663	0.674
E	0.156	0.138	0.138	0.625	0.6	0.618	0.615	0.612	0.624
F	0.209	0.178	0.185	0.415	0.437	0.427	0.472	0.462	0.457
G	0.277	0.264	0.264	0.381	0.384	0.385	0.442	0.561	0.318
H	0.048	0.044	0.045	0.045	0.043	0.046	0.044	0.046	0.043

MCF7
E-Ac1/ E-Ac2

Measurement count: 1   Filter: 570

	1	2	3	4	5	6	7	8	9
A	0.04	0.039	0.041	0.04	0.04	0.04	0.041	0.042	0.04
B	0.088	0.1	0.101	0.724	0.722	0.712	0.718	0.721	0.752
C	0.108	0.101	0.105	0.718	0.696	0.719	0.667	0.663	0.66
D	0.115	0.11	0.098	0.647	0.628	0.626	0.604	0.541	0.551
E	0.134	0.102	0.133	0.335	0.318	0.218	0.133	0.274	0.249
F	0.159	0.159	0.154	0.219	0.187	0.252	0.235	0.24	0.257
G	0.215	0.205	0.201	0.251	0.247	0.247	0.243	0.24	0.243
H	0.042	0.041	0.042	0.042	0.04	0.039	0.04	0.04	0.041

10	11	12
0.039	0.044	0.04
0.103	0.103	0.11
0.117	0.121	0.127
0.136	0.137	0.147
0.174	0.176	0.19
0.244	0.245	0.265
0.319	0.359	0.359
0.041	0.041	0.047

10	11	12
0.04	0.04	0.041
0.101	0.105	0.112
0.104	0.1	0.112
0.112	0.126	0.111
0.137	0.102	0.138
0.163	0.153	0.182
0.216	0.211	0.22
0.04	0.04	0.046

10	11	12
0.04	0.04	0.04
0.099	0.1	0.106
0.115	0.115	0.123
0.134	0.122	0.149
0.152	0.182	0.183
0.231	0.241	0.247
0.065	0.065	0.066
0.041	0.041	0.043

10	11	12
0.04	0.041	0.04
0.099	0.1	0.111
0.097	0.108	0.106
0.114	0.107	0.103
0.122	0.115	0.133
0.158	0.157	0.171
0.168	0.209	0.248
0.04	0.041	0.046

10	11	12
0.041	0.042	0.04
0.099	0.1	0.106
0.117	0.118	0.123
0.145	0.136	0.141
0.166	0.172	0.177
0.233	0.236	0.249
0.332	0.314	0.328
0.043	0.04	0.044

10	11	12
0.041	0.04	0.041
0.11	0.126	0.092
0.1	0.1	0.11
0.139	0.115	0.129
0.122	0.134	0.126
0.128	0.071	0.154
0.201	0.204	0.199
0.041	0.042	0.044

10	11	12
0.04	0.04	0.042
0.099	0.1	0.105
0.115	0.115	0.123
0.132	0.132	0.142
0.162	0.166	0.178
0.232	0.225	0.248
0.359	0.258	0.338
0.045	0.043	0.048

10	11	12
0.041	0.04	0.045
0.1	0.097	0.105
0.11	0.099	0.106
0.104	0.121	0.115
0.119	0.12	0.123
0.138	0.166	0.154
0.201	0.189	0.2
0.041	0.03	0.045
